# Testing the Impact of Variations in Administration on the Kessler Psychological Distress Scale (K10)

**DOI:** 10.1177/10731911241256430

**Published:** 2024-06-05

**Authors:** Miranda R. Chilver, Richard A. Burns, Ferdi Botha, Peter Butterworth

**Affiliations:** 1 National Centre for Epidemiology and Population Health, The Australian National University, Canberra, Australian Capital Territory, Australia; 2 Melbourne Institute: Applied Economic & Social Research, University of Melbourne, Parkville, Victoria, Australia; 3 ARC Centre of Excellence for Children and Families over the Life Course, Indooroopilly, Queensland, Australia; 4 School of Psychology, Deakin University, Burwood, Victoria, Australia

**Keywords:** recall period, depression, anxiety, psychometrics, measurement, bias, self-report

## Abstract

Self-report measures are useful in psychological research and practice, but scores may be impacted by administration methods. This study investigated whether changing the recall period (from 30 to 7 days) and response option order (from ascending to descending) alters the score distribution of the Kessler Psychological Distress Scale (K10). Participants were presented with the K10 with either different recall periods or different response option orders. There was weak evidence of lower mean K10 scores when using a 7-day recall period than when using the 30-day recall period (B = 1.96, 95% CI [0.04–3.90]) but no evidence of a change in the estimated prevalence of very high psychological distress. Presenting the response options in ascending order did not affect mean scores, but there was weak evidence of reduced prevalence of very high distress relative to the descending order (incidence rate ratio [IRR] = 0.60, 95% CI [0.36–0.98]). These findings suggest that varying the administration method may result in minor differences in population estimates of very high psychological distress when using the K10.

Self-report measures are ubiquitous in psychology as they can provide valuable insight into a person’s internal psychological states (Haeffel & Howard, 2010). Despite their widespread use, it is well known that self-report measures are vulnerable to self-report biases, and differences in how self-report measures are administered can impact their results (Bowling, 2005). Changing the recall period, the timeframe a person is to consider when answering the survey, and changing the presentation order of the response options can alter the response distribution of self-report measures (Krosnick & Alwin, 1987; Thomas & Diener, 1990). The Kessler Psychological Distress Scale (K10) is widely used in population studies of mental health (Connelly & Platt, 2014; Ferketich & Binkley, 2005; Gravel & Béland, 2005; Korten & Henderson, 2000) and is sometimes administered with different recall periods (e.g., 7 days instead of 30 days) or with response options presented in ascending instead of descending order. However, it is unclear how sensitive this measure is to these variations. The aim of the present study was to evaluate whether these different methods of administration have an impact on estimates of distress and the distribution of scores.

Altering the recall period may be done to tailor a measure for a particular use case. Shorter recall periods are generally more sensitive to variation in symptoms, especially over shorter timescales, making them ideal for use in clinical intervention studies or for short-term symptom monitoring (Keller et al., 1997). However, shorter recall periods are not ideal for assessing a person’s clinical symptoms as symptoms must be sustained to meet diagnostic criteria (Batterham et al., 2019). Where recall period effects have been observed, scores tend to be lower for shorter relative to longer recall periods (Batterham et al., 2019; Keller et al., 1997; Stull et al., 2009). This likely reflects reduced opportunity to experience a range of symptoms over a longer time span. In addition, a bias toward recalling more negative events over longer recall periods could also contribute to higher reported distress symptoms when using longer recall periods (Sato & Kawahara, 2011; Thomas & Diener, 1990). Although there is some evidence that altering the recall period could impact reported symptoms, our prior investigation of the 6-item Kessler Psychological Distress Scale (K6) items within the current sample found no significant change in mean scores when using the 7-day recall period compared to the 30-day recall period (Chilver et al., 2023). However, the item-level analysis found that there was a significant effect of the recall period on feeling nervous in the K6, suggesting that there could be heterogeneity in how sensitive different items or symptoms are to the changes in recall period. The K10 scale includes four additional items, regarding feeling “tired out,” “so nervous nothing could calm you down,” “so restless you could not sit still,” and “depressed” which have not been tested. In addition, our prior work focused on mean K6 scores and a novel binary outcome indicating high psychological distress but did not examine whether changes in the recall period differentially impact individuals with low scores compared to those with high scores (Chilver et al., 2023). The current analysis aimed to test the widely used K10 which includes the additional four items and to assess whether scores across the full distribution of responses are affected (or not affected) equally by including a quantile regression analysis.

Another consideration for the K10 is the order in which response items are presented, which has been found in some cases to alter response tendencies (Bowling, 2005; Chan, 1991; Krosnick & Alwin, 1987). In the original K10 scale, response items were presented in descending order, from *all of the time* to *none of the time* (Kessler et al., 2002). However, the K10 is regularly used by the Australian Bureau of Statistics (ABS) in a range of population surveys and statistics, including the National Health Survey (Australian Bureau of Statistics, 2017) using an ascending response option order (i.e., from *none of the time* to *all of the time*). The effect of this change on reported K10 scores has not been investigated. Past studies have indicated that when responding to text-based questionnaires, there is sometimes a tendency for respondents to select response items appearing earlier in the presented options, known as a primacy bias (Chan, 1991; Krosnick & Alwin, 1987). This pattern of responding has also been found to correlate with a broad range of other response tendencies that reduce the cognitive load required to respond to questions, referred to collectively as satisficing techniques (Krosnick, 1991; Narayan & Krosnick, 1996). Using an ascending response scale in place of a descending scale could result in lower average scores among respondents more prone to satisficing (Bowling, 2005; Keusch, 2018). Because not all self-report measures are impacted by response item order (e.g., Weng & Cheng, 2000), it is unclear whether this effect would be observed with the K10. Our previous research on the K6 (Chilver et al., 2023) did not examine this question.

The two aims of this study are therefore to (a) evaluate whether the recall period (7 days or 30 days) influences the distribution of K10 psychological distress scores and (b) evaluate whether changing the response order from descending to ascending impacts the response distribution. Due to a lack of a theoretical basis for an interaction between recall period and response option order, we opted to assess these effects separately rather than using a crossed design.

## Methods

### Participants

The participants for this study were sampled from the online research panel of the Australian Online Research Unit (ORU). The ORU recruits panel members from within Australia using both online and offline methods to build a regionally representative panel from the general public interested in research participation. For this study, potential participants were randomly selected from the panel and offered the ORU’s usual incentive for participation. Participants had to be at least 18 years of age and residing in Australia. The ORU aimed to recruit equal numbers of males and females with equal representation across age groups. No exclusion criteria were applied. A power analysis determined that a total target sample size of 500 participants, with 250 participants per group, would provide 80% power to detect a small effect size using a within-person design. Additional participants were to be contacted to allow for non-response. The procedure was approved by the ANU Human Research Ethics Committee (Protocol 2021/736), and the research was conducted in line with the Declaration of Helsinki.

### Measures

This study focused on the K10, a widely used measure of psychological distress that has been validated as a short screening scale for mental illness (Kessler et al., 2002, 2003). It consists of 10 items that ask about the frequency of different depression and anxiety symptoms over the past 30 days using a 5-point response scale ranging from *all of the time* to *none of the time.* This scale uses four items excluded from the K6, which relate to feeling tired out for no good reason, so nervous nothing could calm you down, so restless you could not sit still, and depressed. Regardless of response order, the current study used the same scoring system applied by the ABS where each item is scored from 1 to 5, where 1 indicates *none of the time* and 5 indicates *all of the time.* Using this method, scores on the K10 range from 10 to 50, with scores of 30 or more indicating very high psychological distress (Australian Bureau of Statistics, 2017). The K10 psychological distress scale is widely used in research and clinical practice given that previous research has found that scores of 30 or more correspond with the presence of mental illness (Kessler et al., 2003). The experimental manipulation used a within-person design to examine the effect of the 7-day vs. 30-day recall period (using the standard descending response scale format) and the effect of descending vs. ascending response scale (using the usual 30-day recall period).

### Procedure

Participants were randomly allocated to either the (a) recall period condition or the (b) response item order condition:

1. The participants assigned to the recall period condition completed the K10 with both a 30-day and 7-day recall period and response options in descending order only, consistent with the Harvard version of the questionnaire. The order at which participants saw each recall period was counter-balanced between participants.
2. The participants in the response item order manipulation completed the K10 with both ascending and descending response options with a 30-day recall period only. The order of the response conditions was counter-balanced between participants.

In between the two experimental conditions (i.e., either between the 30- and 7-day versions or between the ascending and descending versions), participants completed an unrelated set of questionnaires on the topic of psychological flourishing, not further discussed here. The distractor task took approximately 7 minutes on average.

#### Statistical Analysis

Effects of recall period and response item order were tested with a series of regression models. Linear mixed models were used to assess whether mean distress scores differed between conditions, quantile regression was used to assess whether those with higher or lower distress scores were affected more by the tested conditions than others, and generalized linear models with log link (binary regression) were used to assess whether these conditions affected the proportion of respondents identified as having very high distress. Each model tested for the main effect of condition, response occasion (referring to the first or second condition presented), and for interactions between experimental condition and response occasion.

#### Data Availability

The data and stata analysis script used in this study have been made publicly available on the Open Science Framework at https://osf.io/aezt6/.

## Results

### Sample Characteristics

The full sample consisted of 660 adults residing in Australia. Five participants were excluded due to missing data, leaving 655 participants in the analysis. Of these, 327 were assigned to the recall period condition, and 328 were assigned to the response item order condition. The age and gender distribution of the final sample for each study condition are provided in Table 1. The table shows there was an approximately equal distribution of age and sex across both conditions, although with some over-representation of those in the 35 to 44 years age range. The sample was well-educated, with 92% reporting they had completed high school or an equivalent qualification and 54.5% reporting they held a graduate degree. Means and standard deviation for the K10 total score and individual items are shown in Table 2 by condition. Mean psychological distress was similar across conditions. Similarly, the prevalence of very high distress was similar between conditions, although slightly lower in the ascending condition than in the descending condition.

**Table 1:** Age and Gender Distribution by Condition.

Age	Recall period (*N* = 327)		Scale order (*N* = 328)	
	Men	Women	Men	Women
18–24	27 (4.1%)	32 (4.9%)	22 (3.4%)	31 (4.7%)
25–34	31 (4.7%)	26 (4.0%)	27 (4.1%)	29 (4.4%)
35–44	39 (6.0%)	29 (4.4%)	27 (4.1%)	40 (6.1%)
45–54	19 (2.9%)	21 (3.2%)	27 (4.1%)	24 (3.7%)
55–64	31 (4.7%)	32 (4.9%)	22 (3.4%)	22 (3.4%)
65+	19 (2.9%)	21 (3.2%)	32 (4.9%)	25 (3.8%)

*Note.* Percentages presented are relative to the total sample.

**Table 2:** Means and Standard Deviations for K10 Total and Items.

	Recall period sample				Response order sample			
	7 days		30 days		Ascending		Descending	
Measure	*M*	*SD*	*M*	*SD*	*M*	*SD*	*M*	*SD*
K10 total	20.4	9.5	20.8	9.4	20.9	9.8	21.1	9.5
Tired out	2.5	1.2	2.6	1.1	2.6	1.2	2.6	1.2
Nervous	2.2	1.1	2.3	1.1	2.3	1.1	2.3	1.1
Nothing could calm down	1.8	1.1	1.8	1.1	1.7	1.0	1.8	1.1
Hopeless	1.9	1.1	1.9	1.1	2.0	1.2	1.9	1.2
Restless or fidgety	2.1	1.1	2.1	1.1	2.2	1.1	2.2	1.1
Could not sit still	1.8	1.1	1.9	1.1	1.8	1.1	1.8	1.1
Depressed	2.1	1.1	2.1	1.1	2.1	1.2	2.2	1.2
Everything was an effort	2.2	1.2	2.3	1.2	2.4	1.3	2.4	1.2
Nothing could cheer up	1.8	1.1	1.9	1.1	1.9	1.1	1.9	1.2
Worthless	1.9	1.1	1.9	1.2	1.9	1.2	1.9	1.2
	*N*	%	*N*	%	*N*	%	*N*	%
Very high psychological distress	71	21.7	70	21.4	66	20.1	76	23.2

### Recall Period

**Figure 1.**
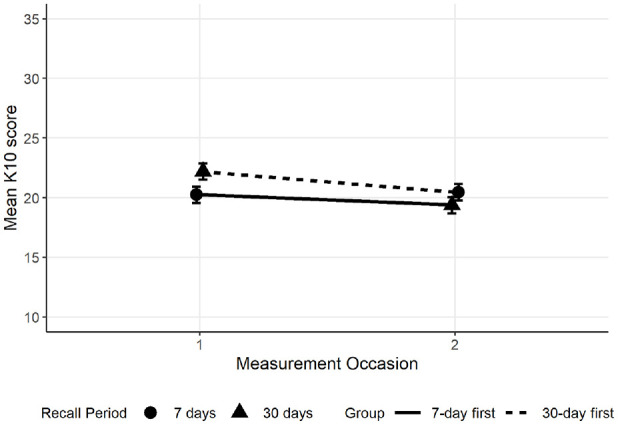
Interaction Between Recall Period and Measurement Occasion. *Note.* Lines indicate groups who completed the 30-day recall condition first (dashed lines) or the 7-day recall condition first (solid line). Scores for the 7-day recall condition are indicated by circles, and scores for the 30-day recall condition are indicated by triangles.

The linear mixed model found scores were 1.96 points lower on average for the 7-day recall period relative to the 30-day recall period (*SE* = 0.97, *p* = .044, 95% CI [−3.87, −0.05]), and scores for the second measurement occasion were lower than the first occasion by 2.83 on average (*SE* = 0.97, *p* = .004, 95% CI [−4.74, −0.92]). While the interaction between recall period and measurement occasion was not significant (*B* = 3.04, *SE* = 1.91, *p =* .111, 95% CI [−0.70, 6.79]), the means shown in Figure 1 suggested a different pattern between recall conditions. Scores from the 7-day recall (indicated by circles) remained similar regardless of whether this condition was presented first or second, but scores from the 30-day recall condition (indicated by triangles) were lower when this condition was presented second. This was confirmed with a follow-up simple effects analysis: A significant decline in distress scores from the first to the second occasion was observed for the 30-day recall condition (*B* = −2.81, *SE* = 0.97, *p* = .004, 95% CI [−4.73, −0.90]) but not for the 7-day recall condition (*B* = 0.23, *SE* = 1.00, *p* = .817, 95% CI [−1.73, 2.20]).

Linear mixed models for each of the individual K10 items showed that only items 1, *tired out for no good reason*, and 9, *so sad nothing could cheer you up*, were scored significantly lower on the 7-day recall period relative to the 30-day recall period. Meanwhile, within the 30-day recall condition, the second measurement occasion was lower than the first for all but three items (so nervous that nothing could calm you down, hopeless, and worthless). There was a significant interaction between recall period and measurement occasion only for item 1, tired out for no good reason, whereby average scores were only lower for the 7-day than for the 30-day recall condition when comparing the first measurement occasion. The simple effects analysis showed again that the effect of measurement occasion was only present for the 30-day recall condition, not for the 7-day recall condition. Full results are provided in the accompanying data file.

**Figure 2.**
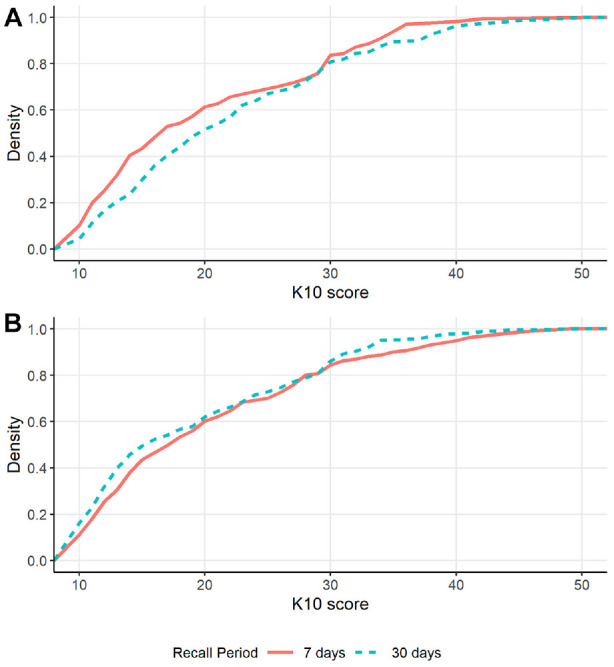
Cumulative Distribution of K10 Scores According to Recall Period on the (A) First Measurement Occasion and (B) Second Measurement Occasion. *Note.* Scores of 30 or higher indicate very high distress.

The K10 cumulative distributions for each measurement occasion are shown in Figure 2. On the first occasion, there was a greater proportion of individuals with scores below 29 in the 7-day recall condition, and also more individuals in the range of 34–36, than in the 30-day condition. The difference in the distribution between the 7-day and 30-day recall periods was reduced on the second measurement occasion, and the pattern was somewhat reversed with more individuals scoring under 19 and more in the range of 31–40 in the 30-day condition relative to the 7-day condition. Quantile regression was used to test for differences between these conditions and potential measurement occasion effects at different points of the distribution. Specifically, we decided *a priori* to test the 20th, 50th (median), and 80th percentiles. These corresponded to scores of 12 (low distress), 18 (moderate distress), and 30 (cutoff between high and very high distress) on the K10 when averaged across conditions.

**Figure 3.**
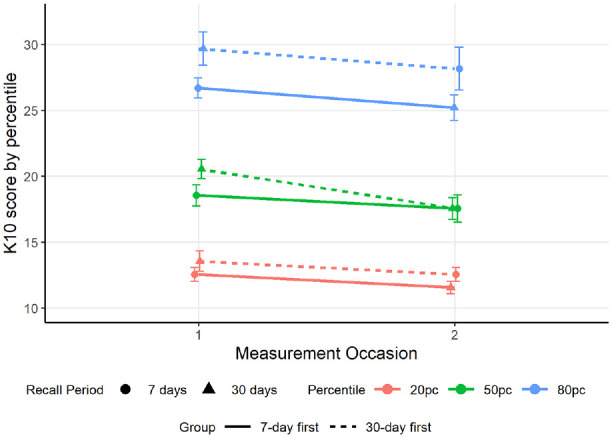
Quantile Scores for 7-Day (Circles) and 30-Day (Triangles) Recall Conditions. *Note.* Lines indicate the response group.

The results showed no difference in the 20th-percentile scores between the 7-day and 30-day condition (*B* = −1.00, *SE* = 0.87, *p* = .253, 95% CI [−2.72, 0.72]). However, the median and 80th percentiles were 2 and 3 points lower for the 7-day relative to the 30-day recall conditions, respectively, (median: *B* = −2, *SE* = 0.90, *p* = .027, 95% CI [−3.78, −0.24]; 80th percentile: *B* = −3, *SE* = 1.44, *p* = .038, 95% CI [−5.84, −0.16]). The 20th, 50th, and 80th percentiles were lower for the second measurement occasion than the first by 2, 3, and 4.5 points, respectively, (20th percentile: *B =*−2, *SE* = 0.91, *p* = .028, 95% CI [−3.78, −0.22]; median: *B* = −3, *SE* = 0.90, *p* = .001, 95% CI [−4.78, −1.22]; 80th percentile: *B =*−4.5, *SE* = 1.58, *p* = .005, 95% CI [−7.6, −1.39]). There was a significant interaction between recall period and measurement occasion at the 80th percentile, indicating that the 30-day recall scores were higher than the 7-day recall period on the first occasion but lower than the 7-day recall period on the second occasion (*B* = 6, *SE* = 2.4, *p* = .012, 95% CI [1.30, 10.70]). Quantile scores are shown in Figure 3.

Given that there was an interaction between recall period and measurement occasion and because most applications of the K10 do not involve repeated measurement in such a short timeframe, we include the results from analyses that include only the between-subjects comparison at the first measurement occasion. When examining only the first measurement occasion, there was still evidence that scores were lower for the 7-day relative to the 30-day recall period (*B* = −1.96, *SE* = 0.98, *p* = .046, 95% CI [−3.89, −0.04]). However, there was no longer evidence of changes in the 20th, 50th, and 80th percentiles (20th percentile: *B =*−1.5, *SE* = 0.85, *p* = .078, 95% CI [−3.17, −0.17]; median: *B* = −2, *SE* = 1.46, *p* = .172, 95% CI [−4.87, 0.87]; 80th percentile: *B =*−3, *SE* = 1.59, *p* = .061, 95% CI [−6.14, 0.14]). When compared to Figure 2A, these tested percentiles do not correspond to the parts of the distribution where the recall effect was most pronounced, which appears to be around the 40th and 90th percentiles.

The binary regression model was only assessed using the first measurement occasion. There was no evidence that the recall period affected the estimated incidence of very high distress, incidence rate ratio (IRR) = 1.03, *SE* = 0.23, *p* = .907, 95% CI [0.66, 1.61].

### Response Item Order

**Figure 4.**
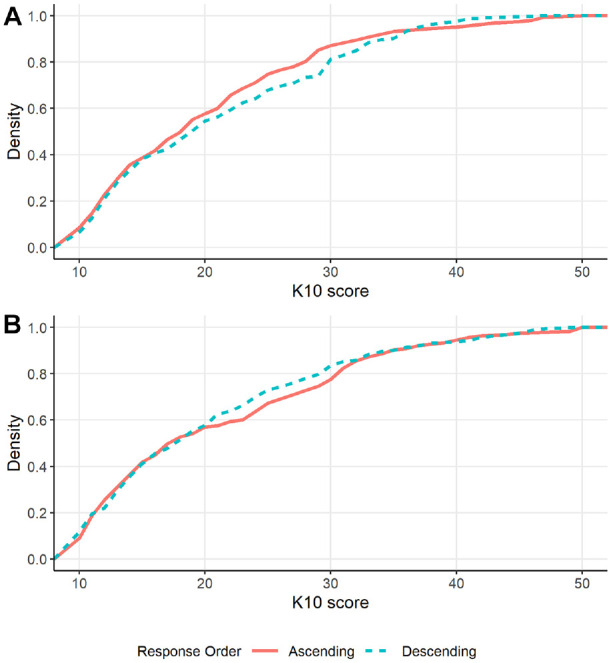
Cumulative Distribution of K10 Scores According to Response Option Order on the (A) First Measurement Occasion and (B) Second Measurement Occasion. *Note.* Scores of 30 or higher indicate very high distress.

The cumulative distribution plots of the response option condition at each measurement occasion are shown in Figure 4. The linear mixed model and quantile regressions found no evidence of an effect of response option order, measurement occasion, or an interaction between order and occasion either for the total scale or for any individual items. This remained the case when tested at the first measurement occasion only. However, the binary regression model provided some evidence that presenting the response options in ascending order resulted in a lower estimated incidence of very high distress than when presented in descending order (IRR = 0.59, *SE* = 0.16, *t* = −1.96, *p* = .050, 95% CI [0.35, 1.00]). Neither the response occasion effect (IRR = 0.81, *SE* = 0.20, *t* = −0.85, 95% CI [0.50, 1.32]) nor the interaction effect (IRR = 2.04, *SE* = 0.79, *t* = 1.85, *p* = .064, 95% CI [0.96, 4.34]) was significant. Limiting this analysis to the first measurement occasion still indicated that the incidence of very high distress was lower when using the ascending response scale than when using the descending scale (IRR = 0.60, *SE* = 0.15, *t* = −2.05, *p* = .041, 95% CI [0.36, 0.98]).

## Discussion

The aim of this study was to investigate whether changing the recall period and response option order of the K10 alter the distribution of K10 scores in the general population. Although it has been assumed that these changes would have minor effects on the interpretation of the K10 (National Comorbidity Survey, 2022), this has not previously been formally tested. Given the wide application of the K10 globally in both clinical and research contexts with subtle differences in administration, it was important to test for recall period and response order effects. The results revealed that using a 7-day recall period instead of the original 30-day recall period led to a small but statistically significant reduction in mean K10 scores but had no effect on the proportion of participants classified with very high distress, although we note that the evidence for this effect is relatively weak (*p* = .044). In comparison, changing the order of response options from a descending (*all of the time* to *none of the time*) to an ascending scale (*none of the time* to *all of the time*) had no impact on mean K10 scores but slightly decreased the incidence of very high distress. These results suggest that subtle differences in the administration of the K10 can result in small changes in self-reported psychological distress in certain populations.

We found that shortening the recall period to 7 days resulted in a small reduction in mean self-reported psychological distress. This is in line with previous findings from other self-report measures indicating that self-reported symptoms tended to be lower for shorter relative to longer recall periods (Batterham et al., 2019; Keller et al., 1997) but contrasts slightly with our previous analysis of the K6, which found no significant effect of recall period (Chilver et al., 2023). This difference may be because recall period significantly affected the responses for the item “tired out for no good reason,” which is in the K10 but not the K6 (in addition to the item “so sad nothing could cheer you up,” which is in both). Although there is some divergence in the results in terms of significance testing, the K10 recall period effect was weak, and the effect size confidence interval overlaps with the K6 findings (CI [−2.17, 0.18] for the K6 compared to CI [−3.87, −0.05] for the K10). Both findings suggest a trend toward lower scores for the 7-day recall period than for the 30-day recall period but indicate a likely small or negligible effect. Further research may benefit from exploring whether certain distress symptoms are more sensitive to different recall periods than others.

Notably, when examining mean scores, there was also a main effect of measurement occasion that was larger than the recall period effect. This raises a potential concern about the test–retest reliability of the K10. However, previous research has found the K10 to have high test–retest reliability (Merson et al., 2021). Furthermore, the effect of measurement occasion on K10 scores was only present in the recall period experiment and did not replicate when testing the effect of response option order, suggesting that this measurement occasion effect in the recall period experiment was either spurious or impacted by the within-person change in recall period. Specifically, distress ratings for the past 7 days appeared to be unaffected by measurement occasion, whereas distress ratings for the past 30 days tended to be lower when the 30-day recall period was presented on the second measurement. Previous research comparing daily with retrospective emotion ratings has shown recall over shorter periods to be more accurate (Thomas & Diener, 1990), which may be why the 7-day recall period was not affected by the repeated measurement, but responses to the 30-day recall period varied. Participants tended to estimate their psychological distress as being lower and more similar to their 7-day estimate when the 30-day recall period was presented second. The more accurate recall of the past 7 days might have had an anchoring effect on responses. Given that most applications of the K10 would not involve completion of both the 7-day and 30-day recall conditions at the same time, we focused on the between-subjects effect at the first measurement occasion.

The current study aimed to investigate the recall period effect in more detail using quantile and binary regression. The quantile regression initially found evidence that the median and 80th-percentile scores were lower for the 7-day recall period than for the 30-day recall period, but there was no difference in the 20th percentile. This suggests that the change in recall period impacted individuals with more symptoms more than it impacted those with relatively few symptoms. This could be because those with low symptoms are already responding with “none of the time” for most items on the 30-day recall and do not have the ability to provide lower responses on these items for the 7-day recall period, whereas high scorers have more range to provide different scores when the recall period changes. Alternatively, there could be certain ranges of symptom levels where respondents are more prone to overestimating their symptoms over longer timeframes, resulting in higher average scores for the past 30 days than for the past 7 days. However, there was an interaction between recall period and response occasion whereby 80th-percentile scores differed based on whether the 30-day recall period was presented first or second, and there was a similar insignificant interaction trend for the mean scores. On the first measurement occasion, there is a between-person effect where people report higher symptoms over the past 30 days than over the past 7 days, consistent with the main effect. There is also relative consistency in reported symptoms over the past 7 days between people regardless of whether they answered the 7-day recall first or second. However, responses for the 30-day recall period differ between people depending on the recall condition order. Individuals who were presented with the 7-day recall period first tended to report a similar level of symptoms to those presented with the 30-day recall period, whereas those who completed the 30-day recall period first tended to report higher symptoms for the past 30 days than for the past 7 days. Past evidence has indicated that emotional recall is more accurate over shorter periods and that people tend to overestimate the intensity of emotional states over time (Thomas & Diener, 1990). It is possible that when the 30-day recall is presented second, participants are more likely to depend on their more recent, more easily recalled symptoms to inform their answer than when they are asked about the past 30 days first. Further experimental research is needed to replicate and understand this interaction effect, although it is noted that repetition of the same questionnaire for different recall periods is infrequently applied in real-world clinical settings.

When the quantile regression was conducted using only the first measurement occasion, there was no longer evidence that the median or 80th-percentile scores differed between recall conditions. However, there remained weak evidence that mean scores were significantly lower for the 7-day recall condition than for the 30-day recall condition. When looking at the cumulative distribution plot for the first measurement occasion (Figure 2A), the largest difference between the distributions occurs around the 40th percentile, corresponding to scores around 14 for the 7-day recall period and around 18 for the 30-day recall period. That is, scores were more likely to be in the low range when the 7-day recall period was used, which may be due to the reduced opportunity to experience infrequent symptoms over 7 days compared to 30 days (Stull et al., 2009). The change in response distribution did not influence the incidence of very high distress, as indicated by the binary regression model. Thus, while there were potentially some slight differences in the distribution, these changes are predominantly among those with low to moderate symptoms rather than at the high end of the distribution. This impact on the distribution is therefore less likely to impact on clinically relevant decisions regarding access to mental healthcare services.

Changing the response option order from a descending to an ascending scale did not change the mean, median, 20th, or 80th percentile scores. However, there was a small but significant increase in the number of individuals with very high distress when the response options were presented in descending order compared to ascending order. While the evidence for this effect was also weak (*p* = .050), these results align with previous research indicating that response order effects tend to be weak in the general population (Weng & Cheng, 2000), but that there may be more pronounced satisficing effects among populations with lower motivation or poorer cognitive ability who may apply satisficing methods to reduce the effort required to complete the questionnaire (Keusch, 2018; Krosnick & Alwin, 1987). Satisficing can take multiple forms, including selecting the first satisfactory response that is presented, selecting the same response repetitively, or picking responses at random (Krosnick, 1991). Individuals with anxiety and major depressive disorder can experience cognitive symptoms that appear to increase the likelihood that they will utilize satisficing techniques (Conijn et al., 2020). This could be a concern when using questionnaires to measure psychological distress as those with greater symptom levels might be more likely to change their response according to the order response options are presented. Further research on this effect particularly in samples with high psychological distress is warranted to clarify if response option presentation order influences whether individuals are classified as having a common mental disorder.

The current study assessed how administrative changes to the K10, specifically changes to the recall period and response presentation order, might impact the distribution of K10 scores. We found a small effect of the recall period whereby self-reported symptoms were slightly lower when rating the past 7 days than when rating the past 30 days, but this effect did not change the likelihood that a person would be identified with very high distress according to the K10 cut point. While changing the presentation of response items from descending (*all of the time* to *none of the time*) to ascending (*none of the time* to *all of the time*) did not influence the distribution of K10 scores overall, there was a slight reduction in the proportion of those identified with very high distress when the ascending scale was used. These findings suggest that varying the administration method may result in minor differences in population estimates of very high psychological distress when using the K10. While the size of the effect appears to be minor, in a large sample, this could lead to small but significant differences in self-reported psychological distress levels due to administrative approach alone. However, large differences in self-reported psychological distress between groups cannot be explained by administrative variation.
